# Society Discussions

**Published:** 1870-09

**Authors:** 

**Affiliations:** Florence, Italy


### "SOCIETY DISCUSSIONS."

FLORENCE, ITALY.

There is one feature attending the social gatherings of the "sons of the forceps," and that is the amount of egotism displayed by persons who love to talk, and by others who are entirely innocent of any wish to stand always in the advance when anything is to be said.

This egotism does not partake so much of the "mighty I," as it shows another feature, and that is, "always proclaiming what the person has done, what he can do, and what he *will* do," yet scarcely ever in all these discussions do we find "information asked."

It does seem indeed singular, that out of a body of fifty to one hundred men, who in every day life encounter all kinds of cases, difficult and simple, each one should always have done just as the case indicated. It is exceedingly singular that no mistakes are made. How is this? You very seldom ask for information, therefore, the natural conclusion is—you don't need it. In presenting this subject to the profession, I propose to make three distinct points as to the excessively bad influence of this manner of hob-nobbing.

*First.* It effects every individual in a moral and pecuniary aspect. No man can forever tell other people how to do every thing, no man can constantly advise friends—never asking advice, without unconsciously drifting into a self-complacency, a blindness in his ideas of his own powers, that are bound to not only at some time or other reflect upon his professional capabilities, but to leave its hurt on the heads of patients. All have their black Mondays. All have their days of failure. All find each day a place to insert an interrogation point. All have black weeks at times. If you can by any possibility delude your self that your public sentiments should be illustrated in the office, as expressed in public, never asking for information, you will some bright day "be brought up standing" when you the least expect it. One good day of honest confession of failures, and a putting of heads together to solve the difficult problem, would do more good than a dozen years of fine scientific discussions. Don't imagine we oppose discussion. What is a convention worth without hearty, healthy, *big* debates? But in your three or four days councils, give one little forenoon to asking information. There are a few that come with that purpose and carry it out. There are many that come with the same purpose in view, and upon honer, they are so frightened out of their wits by the greatness of the apparent never fail minds they find there, that they cringe and crawl off never saying a word. It, as I said effects the pocket.

Men who generally need the most information are those that can illy afford to leave their offices for any length of time. They feel the outlay attendant upon these trips. Some come a long way. And in general, Mr. Editor, what do they hear? They are fairly taken aback that their professional brethren are *always* successful; that no confession of failures and way of escape is ever spoken of. The consequence is these men go home with a vague idea of a "talk," but in many instances without benefit.

One man is benefited by this scientific discussion, another by that elaborately written manuscript. Others need more solid food—and they can't find it.

Again, the effect on young practitioners is bad. How many good honest young Dentists, with families to support, and seemingly with more than their share of ill luck, both in the laboratory and at the chair, attend these meetings, and when they discover that everybody else walks the flag-stones of the profession, patent leathered and kidded—all success—how many leave heart-sick, discouraged, in that they carry on their garments seemingly, all the slough of professional bad luck. I know of some that have actually for this cause given up the business. There is no denying that there is scarcely a profession, that has for its supporters, men who are so jealous of making the acknowledgment—*I am wrong.* It is a fact, and you *all* know it. In Italy, the Dentists, the native Dentists I refer to, have no associations. They are jealous of each other, and don't wish to swap ideas. They know enough and they stay at home.

In America, you all know enough, and you get together and tell it. This is the only difference that I can see in five out of ten of your associations. Dr., if you will edit a magazine in which men will come forward and tell what they don't know, I will pay you ten dollars annually, and I know many that would do the same. I acknowledge this letter is an "orful wail," but if you lived as far from your native land as I do, and when the post man brought you the ever welcome REGISTER or *Cosmos*, you, in your eagerness to see what your brothers "o'er the sea" were doing, find only in half the instances senseless discussions, you would wail too. The third point is, it retards the advancement of Dentistry. The first great impetus received, say twelve or fifteen years ago, was a mighty heave by all together. They were accustomed in those days to give ten clinics, where now they give one. I would give more to see a man like Dr. Allport fill a cavity, or the famed plate workman of Kalamazoo, (I have at this moment forgotten the name,) make a set of teeth, than listen to a week of discussions such as we generally hear when we all get together for one of our quarterly or annual "pow-wows."

